# Montreal Veterinary Medical Association

**Published:** 1897-01

**Authors:** B. A. Sugden

**Affiliations:** Secretary-Treasurer


### MONTREAL VETERINARY MEDICAL ASSOCIATION.

THE regular meeting was held November 19th in the library of the College. The Vice-President, Dr. Charles McEachran, occupied the chair.

There being no business, the chairman called upon Mr. Newcomb to report his case, which was one of "Supernumerary Testicles in the Horse," interesting from its rarity and the denial of its occurrence by several authors who consider mistaken diagnosis responsible for such cases; but Mr. Newcomb, being so confident of a correct diagnosis, had no hesitancy in reporting the following case: On June 16, 1895, he was called to castrate a well-bred two-year old. On examination no sign of scrotal hernia was discovered, but quite a difference in the size of the testicles was noticeable, the left being one and one-half times the size of the right. The colt was cast and the right testicle removed; the left side of the scrotum was then incised, and to his surprise the testicle exposed was no larger than the first; a little traction made the cause of the variation in size apparent. The spermatic cord was divided, and on the branch was a third testicle which did not differ from the other except in size. It possessed an epididymis, and, as far as the naked eye could discern, had the same structure. The ecraseur-chain was passed over the cord above the division and both testicles removed. In May, 1896, he received a letter from the owner asking him to examine the same colt. Dr. E. H. Morris and himself examined the animal and found the right side of the scrotum enlarged and containing what they had no doubt was a testicle, although it seemed firmly adherent to the walls of the scrotum. On the 19th of May they operated and removed another testicle. The covering was inseparable and it was necessary to dissect the testicle away from the scrotum. The cord was thickened and very tough, and, after breaking an ecraseur-chain, the emasculator was used to sever it. The wound healed splendidly and the animal caused no further inconvenience to the owner.

Mr. Cullen followed with a paper on "Horseshoeing." The members present are to be congratulated on having heard an essay presenting so many aspects, both theoretical and practical, upon this important subject. The essayist, after describing the provisions of nature for maintaining the foot in a normal condition when the animal was in its normal state—*i. e.*, on the plains—remarked that in his opinion, owing to these provisions being absent in its domesticated condition, careful attention to the feet from birth upward was indicated. Practical shoeing in regard to the different breeds of horses and the various work required of them was fully discussed, together with the more important anatomical and physiological points, the essayist remarking that in his experience it was seldom that a normal foot appeared at the forge, owing to the lack of the aforesaid attention, consequently he would devote the greater part of his essay to the shoeing of abnormal feet. After condemning the various forms of careless and ignorant shoeing, he clearly indicated the most probable methods that might be employed for their correction. Continuing, he described many points of practical interest in the application of shoes for relieving the lameness consequent upon various diseases, such as corns, sand-crack, etc., showing the difficulty under which the blacksmith labored in being frequently required to apply a shoe for the immediate cure of lameness, even after pointing out to the customer the necessity of prolonged treatment and rest. In the discussion which followed the essayist fully demonstrated his familiarity with the subject, practically and clearly answering the numerous questions which his paper evoked. The main points of interest in this discussion appeared to be, first, as regards the permanent use of the bar-shoe for the production of the necessary pressure on the foot-pad, which Mr. Cullen advocated. Secondly, the query as to whether the application of a hot shoe to obtain a level bearing was injurious or not, the essayist upholding the judicious practice of this method for that purpose.

Dr. Charles McEachran, after complimenting the Society on the splendid manner in which the discussion had been maintained and the essayist on the able way in which he had defended his paper, stated that in his opinion in place of the bar-shoe, though undoubtedly often necessary, a slight pressure exerted between the ground and the foot-pad produced by an open shoe conforming to the natural shape of the foot was preferable. The proceedings then terminated.

A meeting was held December 3d in the library of the College, the President, Dr. Baker, occupying the chair.

Mr. Parker reported an interesting case of "Œsophagotomy in the Cow." Before Mr. Parker arrived at the case, the owner and friends had attempted to remove the obstruction, which eventually proved to be a large potato, by pushing it down with a fork-handle. On Mr. Parker's arrival he found the animal in an extreme condition of tympanites, which he relieved by using the trocar; being unsuccessful in his efforts to remove the obstruction by means of the probing, he decided to operate. This Mr. Parker did under strict antiseptic precautions, and was rewarded by the animal making a good recovery, which took some twenty-eight days, during which time the patient was supported by rectal injections of new milk, slowly administered, and by drenches of oatmeal-gruel. Some discussion followed as to the various methods of treating these obstructions, and it was suggested that in cases similar to the above the animal might be supported by direct injection into the rumen.

The President then called upon Mr. Hillard for his paper. This was one on "Laminitis," which he commenced by pointing out that the common name of "foundered" was based on the assumption that an animal so affected was unable to progress favorably, and thus resembled a ship when disabled. The essayist accurately described the symptoms accompanying the acute, subacute, and chronic conditions of this disease. Among the predisposing causes he cited that of crossing light and heavy breeds, a frequent result of which is an animal with feet too light to support a heavy body. Mr. Hillard then discussed the various methods of treatment, and, in concluding, advocated the application of a stout, wide-webbed bar-shoe. The relative value of hot and cold applications was then discussed by the members, the majority being in favor of the latter. The President then briefly gave the history of some cases of laminitis which had occurred in his own practice. His experience had shown him that for treatment to be successful it is essential that it be resorted to early, and as far as hot and cold applications went he had had equally good results from both. If the animal is bled, it should be done early, and the blood rapidly extracted. He advised the members when called upon to see horses taken suddenly ill and breathing rapidly to always have them moved before making a diagnosis.

The Secretary then read a communication from Dr. Rowat, of Hawaii, with regard to a disease which had recently made its appearance in a herd of cattle, and which he designated "Enzoötic Hæmaturia." This was listened to with much interest, and at the conclusion a hearty vote of thanks was unanimously accorded to Dr. Rowat.

The proceedings then terminated. B. A. SUGDEN,
Secretary-Treasurer.

